# INTRAOPERATIVE COMPUTED TOMOGRAPHY: AN ADVANCED APPROACH FOR VISUALIZATION OF FIXATION MATERIAL IN DISTAL RADIUS FRACTURES

**DOI:** 10.1590/1413-785220253303e287214

**Published:** 2025-12-01

**Authors:** Pedro Henrique Pires, Marcela de Melo Gajo, Matheus Kuffner, Gabriel França Calumby, Caio Caldas Couto

**Affiliations:** 1 Brazilian Hand Surgery Society, Sao Paulo, SP, Brazil.; 2 Felicio Rocho Hospital, Minas Gerais, MG, Brazil.

**Keywords:** Distal Radius Fracture, Fluoroscopy, Osteosynthesis, Fracture, Fratura Distal do Rádio, Fluoroscopia, Osteossíntese

## Abstract

**Objective:**

To compare the efficacy of 2D fluoroscopy with intraoperative computed tomography (CT) in detecting intra-articular screws that extend beyond the dorsal cortex in distal radius fractures.

**Methods:**

Prospective study of 10 patients undergoing osteosynthesis of distal radius fractures, evaluating the accuracy of 2D fluoroscopy and intraoperative CT.

**Results:**

2D fluoroscopy did not identify inadequate positioning, while intraoperative CT detected 20% of intra-articular screws and 60% of screws going beyond the dorsal cortex.

**Conclusion:**

Intraoperative CT is more effective in detecting inadequate positioning of the synthesis material and may prevent future complications. *Level of Evidence III; Prospective* ^d^ *comparative study* ^e^.

## RESUMO

**Objetivo:**

Comparar a eficácia da fluoroscopia 2D com a tomografia computadorizada (TC) intraoperatória na detecção de parafusos intra-articulares e que ultrapassam a cortical dorsal em fraturas de rádio distal.

**Métodos:**

Estudo prospectivo com 10 pacientes submetidos à osteossíntese de fraturas de rádio distal, avaliando a precisão da fluoroscopia 2D e da TC intraoperatória.

**Resultados:**

A fluoroscopia 2D não identificou posicionamentos inadequados, enquanto a TC intraoperatória detectou 20% de parafusos intra-articulares e 60% de parafusos ultrapassando a cortical dorsal.

**Conclusão:**

A TC intraoperatória é mais eficaz na detecção de posicionamentos inadequados do material de síntese, podendo prevenir complicações futuras.*Nível de Evidência III; Estudo* ^d^ *prospectivo comparativo* ^e^.

## INTRODUCTION

Fracture of the distal radius is the most common fracture of the upper limb in adults, accounting for 1/6 of all fractures treated in the emergency room. Its anatomical reduction, especially in terms of preserving the intra-articular of the intra-articular surface, is considered essential for maintaining the functionality of the limb. Considering this factor, the use of volar locking plates has been chosen as the best method of osteosynthesis for this type of fracture, especially in the presence of a dorsal fragment with an intra-articular trace and in cases of high comminution.^1,2^ However, the use of locking plates on the volar aspect of the distal radius is associated with a greater risk of the screws penetrating the radiocarpal joint and being positioned beyond the dorsal cortex of the radius. Specifically in comminuted fractures with intra-articular involvement, the synthesis material is positioned more distally, guaranteeing the reduction and stability of the fracture due to its subchondral location.^3^ Currently, intraoperative fluoroscopy is used to check this positioning, but due to the biconcave articular surface of the distal radius, it is not always effective.^1^ Even with the use of this synthesis material, the average post-operative complication rate for distal radius fractures is 16.5%, related to conditions such as osteoarthritis and extensor tendon rupture. Considering the intra-articular protrusion of screws as a causal factor for complications such as osteoarthritis, the use of intraoperative computed tomography (CT) has been considered as an alternative for more reliable detection of the ideal positioning of the synthesis material.^3^ The aim of this study is to compare the use of 2D fluoroscopy with the use of intraoperative computed tomography, in order to detect which imaging method guarantees the most accurate detection of malpositioning of the synthesis material. Specifically with regard to intra-articular positioning and dorsal protrusion of screws, the aim is to assess which imaging method detects these alterations better, in order to avoid future complications related to chondral injury and extensor tendon injury.

## MATERIAL AND METHOD

**Figure 1:**
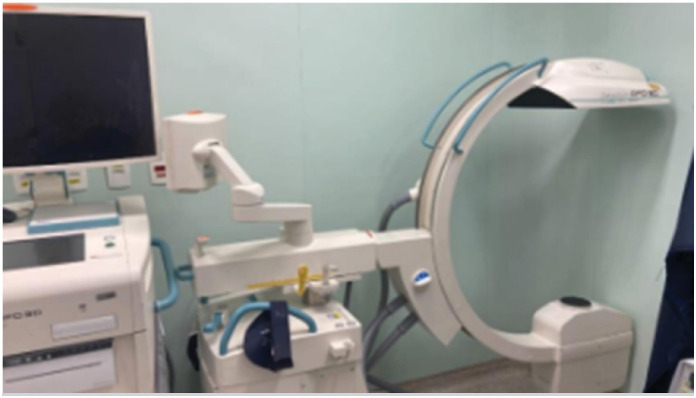
Study flowchart.

This study included patients undergoing surgical treatment for distal radius fractures at Hospital X from January to December 2023. This prospective comparative diagnostic study was approved by the Institutional Review Board/Ethics Committee (CAAE 75178723.4.0000.5125). All participants provided written informed consent prior to enrollment. Patients undergoing osteosynthesis with locked plates and screws were included. Patients treated with Kirschner wires and those with previous signs of radiocarpal joint degeneration were excluded. After selecting the patients, they underwent osteosynthesis of their respective fractures according to prior preoperative planning. The techniques used in the surgery and the method of checking the reduction and positioning of the synthesis material were not altered to suit the study. In other words, during the intraoperative period, the presence of an intra-articular screw was delimited by the 2D fluoroscopic view, using the usual AP and Profile views, as well as the additional skyline view and oblique view with a 30º elevation of the forearm. After completion of the reduction and placement of the synthesis material, the approached wrist was submitted to computed tomography while still in the intraoperative period (Figure 1). The images were analyzed immediately by the attending surgeon to determine the presence of intra-articular material or material extending beyond the dorsal cortex that had not previously been detected by conventional fluoroscopy. Depending on the results obtained with the new imaging method, the surgical procedure was completed or the positioning of the synthetic material was adjusted. The results of the CT scan did not change the definition of which synthesis material would be used or the technique used to implant this material, only the spatial orientation of the screws in relation to the radiocarpal articular surface and the dorsal cortex. Post-operative follow-up of the selected patients was carried out according to the usual pattern of the attending surgeon, weekly for the first two post-operative weeks and then at one and three months after surgery.

## RESULTS

During the study period, 10 patients who underwent surgical treatment for distal radius fractures at Hospital X were included. The average age was 64 years, with a predominance of females (66%). The most common mechanism of trauma was falling from height, and the most common fracture was type C1, according to the AO classification (Table 1). With regard to the side affected, 58% of the fractures occurred on the right side, which was the dominant side. All the patients were treated surgically with locking plates, and 70% of the procedures were carried out less than a week after the fracture.

**Table 1:** Patients’ epidemiologic data.

Patients	Age (Years)	Side	Fracture classification (AO)
1-	39	D	C1
2-	57	D	C3
3-	65	D	B2
4-	76	D	A3
5-	77	E	C1
6-	65	E	B3
7-	46	D	A3
8-	63	D	C1
9-	56	E	C1
10-	29	D	C1

When analyzing the images obtained with conventional two-dimensional fluoroscopy, it was observed that this method did not identify cases of improper positioning, whether intra-articular or exceeding the dorsal cortical, representing 0% in both cases. In contrast, intraoperative CT performed after the final radiographic images presented a 20% identification rate for improper intra-articular positioning (2 out of 10 patients) and a 60% detection rate for screws exceeding the dorsal cortical of the radius (6 out of 10 patients). The higher percentages obtained with this method suggest that CT has superior capability in identifying improper positioning of the fixation material, especially those involving the dorsal cortical of the radius. Among the cases where intra-articular material was verified, 50% occurred in patients with fractures classified as AO type C. Regarding the identification of material exceeding the dorsal cortical, 66% were verified in fractures classified as AO type C (Table 2).

**Table 2:** Obtained results with different image methods.

Patients	Age (Years)	Fracture classification (AO)	Intraoperatory 2D fluoroscopy		Intraoperatory CT	
			IA	DC	IA	DC
1-	39	C1	N	N	N	Y
2-	57	C3	N	N	N	Y
3-	65	B2	N	N	N	Y
4-	76	A3	N	N	Y	N
5-	77	C1	N	N	N	Y
6-	65	B3	N	N	N	N
7-	46	A3	N	N	N	Y
8-	63	C1	N	N	Y	N
9-	56	C1	N	N	N	Y
10-	29	C1	N	N	N	N

Subtitle: IA – intra articular material. DC – material positioned beyond the dorsal cortical. N – No mispositioned material. Y – detected mispositioned material.

## DISCUSSION

**Figure 2:**
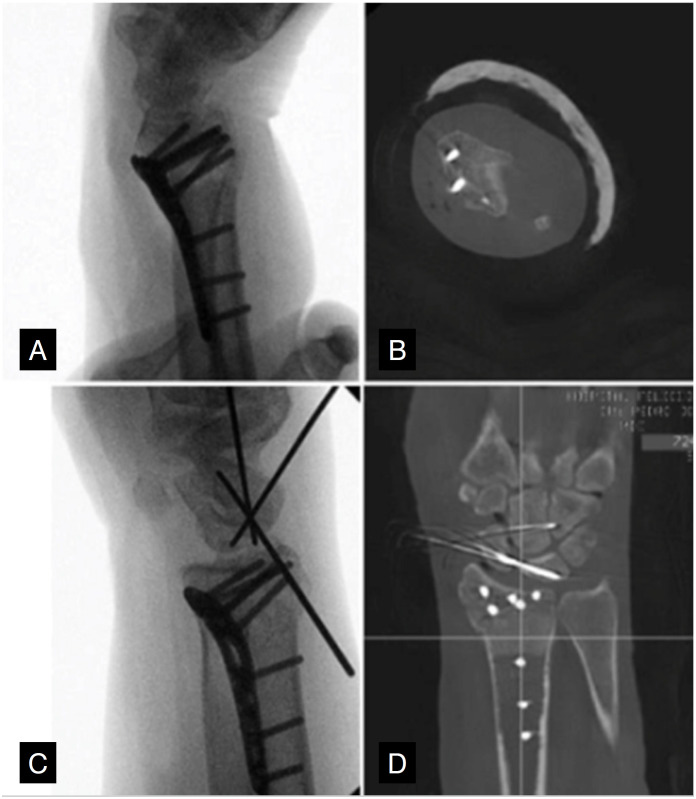
Intraoperative images obtained during the study.

Source: Author. A and B: 2D and 3D fluoroscopy of the same patient, showing of a screw passing through the dorsal cortex by CT. C and D: 2D and 3D fluoroscopy of the same patient, showing detection of intra-articular screw by CT.

Despite representing a major advance in the treatment of distal radius fractures, the use of the volar locking plate as a synthesis material brings with it the need for reliable imaging methods in order to establish the best positioning of the implant and avoid future complications. Especially in fractures consisting of small subchondral fragments, dorsal fragments or large comminution, the positioning of the screws in order to achieve adequate reduction has to be carried out in risk areas, resulting in a greater likelihood of them entering the radiocarpal intra-articular space and exceeding the limits of the dorsal cortex of the radius. There are studies in the literature that advocate the use of unicortical locking screws in the most distal portion of the radius, however, especially in fractures that presente comminution of the dorsal cortex, there is still room for debate as to whether the use of shorter screws determines the same effectiveness for fracture stability.^4^ Considering these factors, there is currently a search for the most appropriate imaging method for detecting inadequacies in the synthesis material in question. The use of traditional 2D fluoroscopy views has long been the most widely long been the most widely used imaging method to confirm the positioning of the plate and screws in the distal radius. However, due to the irregular anatomy of the and dorsal surface of the radius, these images give the false impression that the material is not going beyond the determined limits.^3^ In order to improve this visualization, several additional views have been proposed, the most widely used currently being the Dorsal Tangential View (DTV).^5^ When evaluating 30 fractures of the distal radius, Ganesh et al.^5^ observed that the DTV identified 26.7% of prominent screws in addition to those identified by conventional views. However, post-operative CT scans performed on the same patients identified that 2.86% of the screws remained prominent, even after intra-operative assessment with an additional fluoroscopic view. An interesting aspect of the study was that 80% of the screws not detected by DTV were positioned in the topography of the second extensor compartment. With the same aim of evaluating the efficacy of conventional fluoroscopy, Özbek et al.^4^ compared the use of traditional views with the skyline view in 52 patients with distal radius fractures. In the post-operative evaluation with CT, it was found that the group who underwent the special fluoroscopic view had significantly fewer (p<0.05) prominent screws in the dorsal cortex when compared to the group who underwent the conventional views. Even though this advantage was demonstrated, the use of the skyline view still proved to be flawed since 26.9% of the patients had prominent screws identified by CT scan, most of which were located on the ulnar side of Lister's tubercle.^4^ The proposed use of intraoperative CT scans analyzed in this study seeks to ensure the most effective detection of prominent screws in the dorsal cortex of the radius or screws invading the intra-articular space (Figure 2). Dorsal prominence of the synthesis material is believed to be associated with irritation and rupture of the extensor tendons, a complication seen in up to 50% of patients undergoing surgical treatment for distal radius fractures.^6^ According to the cadaveric study by Austin et al.^6^ the 2-millimeter dorsal prominence of the synthesis material can cause tendon irritation, especially when in the ulnar column of the radius. In the study by Cha SM et al. the follow-up of 314 patients with island fracture of Lister's tubercle, it was concluded that other factors such as the formation of bone callus in the region of Lister's tubercle may also be involved in the rupture of the extensor pollicis longus after surgical treatment of a distal radius fracture.^7^ Despite the small sample size, the results of this study are compatible with those found in the literature. The detection of prominent material in the dorsal cortex in 60% of the fractures previously assessed by conventional fluoroscopy indicates that intraoperative CT is a more effective imaging method for the object in question. With a similar conclusion and a larger sample size, Schnetzke et al. after subjecting 307 distal radius fractures to intraoperative CT identified a 17.6% rate of abnormalities not detected by conventional fluoroscopy.^8^ Considering that in all of these procedures the surgeon considered the positioning of the synthesis material to be optimal after analyzing the 2D image, the revision procedures carried out after the abnormality was identified by CT would not have been carried out if it hadn't been for the use of a more effective imaging exam during the intraoperative period. According to Selles et al. the use of intraoperative of intraoperative CT did not show statistically significant superiority in terms of fracture reduction, but it did indicate the need for intraoperative revision of synthesis material positioning in 11% of patients.^9^ In keeping with the results presented, several studies comparing the use of 3D and 2D imaging in the intraoperative period have achieved synthesis material abnormality detection rates of 17.6% to 32.4% with the use of CT.^2,8,10,11^ In all of these studies, the detection of screws both in the radiocarpal joint and extending beyond the dorsal cortex of the radius resulted in the procedure being revised at the same surgical time, avoiding the need for future re-approachment. According to Mehling et al. the more complex the fracture the more complex the fracture approached, the higher the revision rate after using intraoperative CT, as was observed in their study with a revision rate of 32.4% and detection of 58.8% of the screws in the radiocarpal joint. In this way, the use of intraoperative CT provided a lower risk when extremely distal positioning of screws was necessary, thus making it possible to immediately correct the positioning of the synthesis material.^11^ The debate on the use of intraoperative CT involves its main disadvantages, such as increased radiation and surgical time. In their retrospective comparative study, Halvachizadeh et al.^2^ evaluated 187 patients and detected a radiation exposure of 6.9mGy in the group undergoing intraoperative CT and 2.8mGy in the group undergoing conventional fluoroscopy. In the prospective study by Mehling et al.^11^ the radiation dose per area was calculated at around 3.2 cGycm2, which represented an increase of 55.6% in radiation when compared to the use of conventional fluoroscopy alone. Despite the significant increase in patient radiation, the doses observed are lower than the dose emitted during a Computed Tomography session outside the surgical environment (0.2mSv). The various studies found in the literature are based these data and conclude that the cost-benefit is valid, since the use of intraoperative CT prevents the use of postoperative CT and the submission of the patient to additional imaging exams to monitor the synthesis material. In theanother relevant finding was that the entire radioactive dose to which the patients were subjected, including conventional fluoroscopy, intraoperative and postoperative CT, reached a maximum of 0.25mSv, which is classified as a low-risk dose by the International Commission on Radiological Protection.^2,9-11^ With regard to the increase in surgical time, obtaining and processing intraoperative CT takes an average of six minutes, depending on the familiarity of the with the instrument.^2,8,11^ Considering that it may be necessary to review the procedure after obtaining the image, the total surgical time can increase by up to 28 minutes, or 37% more than the usual time.^10^ As the service and the team adapt to the use of intraoperative CT, the time added to the procedure decreases. the total surgical time is the surgeon's experience and not the type of imaging method used.^2^ The cost of adding this new imaging method as the method of choice for intraoperative distal radius fracture conferencing should also be considered. The cost of acquiring and maintaining these devices is around twice that of traditional fluoroscopy. However, when evaluating the cost-benefit ratio, it should be borne in mind that the use of intraoperative CT prevents the need for future revision surgeries and the need for postoperative CT scans, thus reducing the likelihood of additional costs for the patient.^11^ Hüfner et al. propose that despite the high cost, if used with high frequency and culminating in an intraoperative revision rate of over 5%, the addition of CT to the intraoperative conference protocol would bring economic benefit to orthopaedic services.^12^ The results presented both in this study and in the literature review show that the use of intraoperative CT in the treatment of distal radius fractures ensures better visualization not only of the fracture but also of the positioning of the synthesis material. Its inclusion as the imaging method of choice results in higher revision rates within a timely surgical timeframe and with little radioactive risk for the patient and team. Especially in cases of comminuted fractures with very distal or dorsal fragments, CT becomes an extremely useful tool in detecting intra-articular or prominent screws, thus avoiding future re-approachment and possibly interfering with the rate of post-operative complications.^8^ This study has some limitations, the main one being the small number of patients included. Even with a small sample, the results mirrored those found in the literature, although the absence of true positives made it impossible to calculate variables such as sensitivity and specificity. It should also be considered that the descriptive analysis does not allow us to gauge that the differences presented have statistical significance, but rather suggests that the hypotheses raised are probable.

## CONCLUSION

Intraoperative computed tomography identified abnormalities in the positioning of the synthesis material in 60% of cases. In line with the literature reviewed, the conclusion is supported that there is significant support for the application of this imaging method in order to improve the surgical treatment of distal radius fractures, especially in cases characterized by distal and dorsal comminution. Future studies should further investigate the clinical benefits and challenges associated with this technique.
